# An Unsupervised Deep Hyperspectral Anomaly Detector

**DOI:** 10.3390/s18030693

**Published:** 2018-02-26

**Authors:** Ning Ma, Yu Peng, Shaojun Wang, Philip H. W. Leong

**Affiliations:** 1 Department of Automatic Test and Control, School of Electrical Engineering and Automation, Harbin Institute of Technology, Harbin 150080, China; maning@hit.edu.cn (N.M.); pengyu@hit.edu.cn (Y.P.); 2 School of Electrical and Information Engineering, The University of Sydney, Sydney 2006, Australia; philip.leong@sydney.edu.au

**Keywords:** hyperspectral image, deep learning, anomaly detection

## Abstract

Hyperspectral image (HSI) based detection has attracted considerable attention recently in agriculture, environmental protection and military applications as different wavelengths of light can be advantageously used to discriminate different types of objects. Unfortunately, estimating the background distribution and the detection of interesting local objects is not straightforward, and anomaly detectors may give false alarms. In this paper, a Deep Belief Network (DBN) based anomaly detector is proposed. The high-level features and reconstruction errors are learned through the network in a manner which is not affected by previous background distribution assumption. To reduce contamination by local anomalies, adaptive weights are constructed from reconstruction errors and statistical information. By using the code image which is generated during the inference of DBN and modified by adaptively updated weights, a local Euclidean distance between under test pixels and their neighboring pixels is used to determine the anomaly targets. Experimental results on synthetic and recorded HSI datasets show the performance of proposed method outperforms the classic global Reed-Xiaoli detector (RXD), local RX detector (LRXD) and the-state-of-the-art Collaborative Representation detector (CRD).

## 1. Introduction

An HSI anomaly target is generally defined as a pixel or object which has lower occurrence probability than the background [1]. Examples include pixels from a burning tree in a forest or oil spills in the sea. Utilizing hundreds of very narrow and continuous spectral bands and spatial information, HSI can better discriminate between different types of objects than conventional video imaging in applications including disaster monitoring, defense applications, and food manufacture.

In this paper, we address the problem of anomaly detection of HSI images. Training techniques for HSI anomaly detectors can be divided into the supervised and unsupervised categories. While both have their advantages, we focus on the unsupervised anomaly detectors for the following reasons:

1. Supervised training requires labeled training data, which are not always available.
2. Due to spectral changes caused by weather conditions, camera noises, and temperature, the training set may not in practice be representative of the scene [2,3].
3. Pre-processing techniques to perform data correction and compensation [4,5], which are required in supervised detectors, may affect real-time performance.

Most of the traditional unsupervised HSI anomaly detectors (AD) require the under test HSI to satisfy some distribution assumptions, such as a multivariate Gaussian distribution. While for some under test HSI in real applications, the deviation from the distribution assumption may cause false alarms. Another issue is anomalous pixels contamination which widely exists in local HSI AD. Local HSI AD can perform better than global HSI AD methods in general. In local HSI AD, the anomalies are identified by the distances between under test pixel and the local pixels which are background (normal) pixels in an ideal situation. However, in real anomaly detection, anomalous pixels are usually mixed into some of the local pixels, and lead to the false alarm. This situation is named as anomalous pixels contamination.

To mitigate aforementioned two adverse factors, a new anomaly detector based on adaptive weights and DBN coding is proposed. A DBN based auto-encoder is used to extract the high-level features and the reconstruction errors of HSI without distribution assumption required. The image pixels are represented as a series of short codes generated by the network. By computing the distance between an under test pixel and its neighboring pixels, the anomalous pixels can be determined. To avoid anomalous pixels contaminations, adaptive weights are proposed to describe the different contribution of each neighboring pixel. These weights are adaptively generated from the reconstruction error of each pixel in proposed principle. The main contributions of this paper are summarized as follows:

1. The technique of weighted coding for HSI anomaly detection using DBN is proposed for the first time.
2. An effective statistical weight update technique is proposed to adaptively generate the neighbor weights.
3. To the best of our knowledge, the results reported achieve the highest accuracy to date.

The rest of paper is organized as follows. Section 2 gives a review of the previous literature. Section 3 describes the details of the adaptive weight DBN HSI anomaly detector. Section 4 presents the experiments and results analysis, which is followed by concluding remarks and future works in Section 5.

## 2. Literature Review

Over the past thirty years, the most widely studied methods on hyperspectral anomaly detection are Gaussian multivariate distribution based detectors, such as the Reed-Xiaoli detector (RXD) [6], Local Reed-Xiaoli detector (LRXD) [7] and uniform target detector (UTD) [8]. RXD was proposed in 1990 and is based on the Mahalanobis distance and the assumption that the background follows a Gaussian distribution. A covariance matrix is constructed by using the whole scene background spectral information. Then the anomalies can be determined according to the distance between under test pixels and the background pixels. The method is named Global Reed-Xiaoli detector [6,7]. While in some situations, the Gaussian assumption may not well meet of the whole image, a slide window is used to select part of the background pixels to compute the covariance matrix in RXD; this method is named the Local Reed-Xiaoli detector (LRXD). However, the Gaussian assumption may not be accurate in many scenarios, which directly raised the false alarm rates. Despite this disadvantage, RXD is still widely used as the baseline in HSI anomaly detector studies. To deal with the high dimensionality and the non-linear characteristics of HSI data, Kwon [9] proposed the Kernel RX algorithm, which mapped the data into high-dimensional space to facilitate classification. This technique requires a large amount of computation. To better model the complex HSI backgrounds, Guo [10] proposed a weighted-RXD (W-RXD) and a linear filter based RXD (LF-RXD ) to modify the contribution of each background samples and reduce the adverse impact by anomalous pixels or noisy pixels. This detector achieved a good performance. In addition, there are many variants of the RXD algorithm, such as the subspace-based RX algorithm, local adaptive iterative RX algorithm, weighted RX algorithm and real-time RX algorithm [7,11]. The above RXD based HSI detectors may own high false alarm rate when real images do not exactly follow the distribution assumption.

To avoid the problems of making the Gaussian distribution assumption, Banerjee [12] proposed a support vector data description (SVDD) based HSI anomaly detection algorithm in 2006. In this algorithm, the minimum spherical estimation by the support area of the training data is used to find the anomalous pixels. Khazai [13] proposed an adaptive method to further improve the accuracy of SVDD-based anomaly detector.

More recently, Yuan [14] proposed a local sparsity divergence detector which assumes that the background and target pixels belong to different dictionary subspaces, and the anomalous pixel cannot be well represented by the background dictionary. To further improve the accuracy of the sparse-based detector, Cheng [15] proposed a subspace sparse representation based anomaly detector with an optimized fuzzy C-mean clustering. To model the background with anomalous pixels involved, a collaborative representation HSI anomaly detector (CRD) was proposed [16], which achieves the state-of-the-art performance in HSI anomaly detection.

To well represent the background and anomalous distribution features, topology-based anomaly detector [17] was proposed which builds a graph for connecting close pairs of points. The largest graph component is measured as background points, then using the distances between background and the other pixels to discriminate anomalousness. As the sensor resolution improves, graph-theoretic techniques are used in the anomaly detection to process the complex clutter impact for better detection accuracy [18]. Considering the data redundancy in high spectral resolution, feature extraction, and manifold learning [19,20] was leveraged to project the image points into manifold space. Due to anomalous samples having less effect on the learned manifold model, its projection errors are higher than that of the background points, and then, the anomalousness can be discriminated by the projection errors. Depending on such basic and efficient principle, Olson [21] studied a framework with manifold learning(such as kernel principal component analysis [22]) to realize unsupervised anomaly detection for reducing computation and promoting the detection accuracy. Ziemann and Messinger proposed hyperspectral targets detector with an adaptive version of locally linear embedding [23,24] based on graph theory and manifold approach to separate the target data from the background data, and reach a better detection performance.

Recent progress in applying deep learning to image recognition has introduced new techniques for feature extraction, allowing the spectral and spatial distributions HSI data to be better captured [25,26]. In 2016, deep belief networks (DBNs) were used to reduce dimensionality and extract the high-level features in unsupervised training. A one-class SVM was then applied to achieve anomaly detection [27]. To extract spatial and spectral information for better classification accuracy, a Bi-CLSTM (Bidirectional Convolution Long Short-Term Memory) network based hyperspectral feature leaning method was proposed [28]. For anomaly detection, a DBN based geochemical anomaly detector was proposed [29] considering the fact that anomaly samples occur with a lower probability than background samples, and contribute less to the training of the DBN model. Reconstruction errors of anomaly targets are usually higher than those of background samples. Using this idea, a DBN based hyperspectral anomaly target detector (DBN-AD) was proposed with a DBN based auto-encoder. The reconstruction errors between the input pixels and the output of the DBN auto-encoder are computed as anomaly score [30] directly. The idea is similar to manifold learning based anomaly detection approach [21]. To generate sufficient samples for deep learning in HSI anomaly detection, a transferred convolutional neural network (CNN) was proposed [31], which utilizes reference labeled samples to generate the training dataset. The anomaly targets are measured by the similarity to the output of the CNN.

To summarize, the accuracy of HSI anomaly detectors are mainly influenced by the background distribution assumption and the anomalous pixel contamination effect. Although different approaches have been proposed, the accuracies are still not satisfying. Inspired by the idea of deep learning and weighted RXD, we propose a DBN HSI anomaly detector which needs no background distribution assumption and reduces the influence of anomalous pixel contamination effect by adaptive weights.

## 3. Proposed Adaptive Weight DBN Based HSI Anomaly Detection

It is straightforward that relieving the distribution assumption requirement of HSI AD can improve the detection accuracy, especially in real applications. Thus, in this research, we adopt the DBN models which can effectively learn features from datasets with unknown distributions. Thus, the HSI datasets distribution assumption can be avoided. The DBN model with auto-encoder structure is employed to get the image code which contains HSI features and to get reconstruction errors in an unsupervised way.

Moreover, to decrease the effect of local anomalous pixels contamination, an adaptive weight strategy is proposed by allocating small weights to the anomalous pixels which are wrongly regarded as background pixels. This is based on the fact that anomaly pixels which are less than background pixels contribute less in the DBN model. Thus, their reconstruction errors are usually larger than background pixels.

By combining DBN model and the adaptive weights idea, and inspired by W-RXD [10], we proposed an adaptive weight DBN HSI anomaly detector for better detection accuracy. The following part will first brief the basics of DBN and then present our proposed method.

### 3.1. Deep Belief Network as an Auto-Encoder

**Figure 1:**
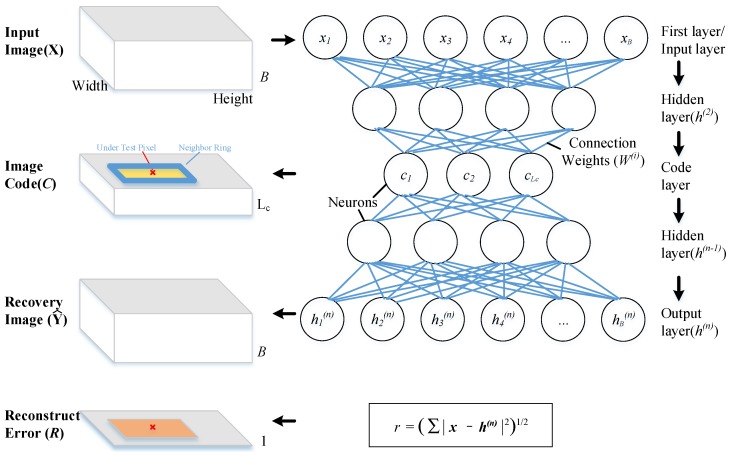
Auto-encode deep learning model structure and data relationship with HSI dataset. Windowheight and Windowwidth stand for the height and width of the input image respectively. $L_{c}$ stands for the length of the code. *B* stands for spectral band numbers. *r* denotes the reconstruction error of each pixel, r∈R. *n* is the number of network layers. i∈[1,n],i is a integer.

A deep belief network is a generative graphical model which is used as an auto-encoder [32]. It models the distribution of the HSI data in an unsupervised way as it is trained to perform an identity operation. Considering the following advantages, DBN is used to learn the features of the image. Firstly, this approach does not impose any assumptions on the distribution of the data. Secondly, the pixels in HSI are encoded into shorter and fixed length codes so that the distance between each code can be measured easily. Thirdly, DBN can learn to probabilistically reconstruct its inputs and to extract a deep hierarchical representation of the training data [33]. With the benefit of multiple non-linear transformations, DBN can perform high-level representation capturing to improve the ability to find the underlying regularities in the data [34,35,36]. These are useful for the processing of the high dimension and non-linear data of a hyperspectral image. Finally, the reconstruction error of each pixel is related to its occurrence probability in the hyperspectral image, which is important for mitigating the anomalous pixels’ contamination. The structure of auto-encoder neural network model and its input–output relationship with HSI dataset are shown in Figure 1.

The model is constructed with one input layer, several hidden layers and one output layer. Different spectral band data of each pixel in Input Image(*X*) are fed to the neurons in the input layer. Only one pixel is input to the network at one time. All the bands of the input pixels are fed to the input layer of the same neuron network. Each layer acts as a function h=f(x;θ) to map the inputs to outputs by several neurons, where the parameters θ can be generated by model training with the input image. The inputs to each layer (except the first layer) are the previous output results multiplied by the connection weights. The neurons number of the output layer is the set to the same with the input layer, and the outputs are regarded as the Recovery Image ($\hat{Y}$). In general, a feedforward neural network can be described by the following formulas.

(1a) $$h^{\left(1\right)}=g^{\left(1\right)}\left(W^{(1)T}x+b^{\left(1\right)}\right)$$

and

(1b) $$h^{\left(i\right)}=g^{\left(i\right)}\left(W^{(i)T}h^{\left(i-1\right)}+b^{\left(i\right)}\right)$$

where i∈[1,n] is the layer order of the network, *i* is an integer, *n* is the number of network layers, $h^{\left(i\right)}$ represents the output of the *i*-th hidden layer, $g^{\left(i\right)}\left(\cdot \right)$ represents the active function in *i*-th layer, $W^{\left(i\right)}$ denotes the connection weights between the i−th layer and the (i−1)-th layer, $b^{\left(i\right)}$ denotes the bias of *i*-th layer neurons, and $W^{\left(i\right)}$ and $b^{\left(i\right)}$ are figured out by training.

A deep learning model is built up by stacking several neuron layers. The optimum level and size of the neuron layers can be determined by grid search methods [37]. The DBN network in the proposed detector is built up with three layers, the neurons number in first and last layers is the same with the spectral number of input HSI dataset and the neurons number of code layer is set to 13. After training using gradient descent [38], this model can describe the mapping from input to output. To encourage high-level sparse features of the dataset, an L1 constraint [32] is used. The cost function is:

(2) $$J\left(\theta ;X,y\right)=J{\left(\theta ;X,y\right)}^{*}+\alpha \Omega \left(h\right)$$

where α∈[0,∞) is the sparsity penalty parameter. In general, *y* is the label of the dataset. In this paper, vecause the network is trained in an unsupervised way, *y* will be replaced by *x*. $\Omega \left(h\right)={∥h∥}_{1}$. $J{\left(\theta ;X,y\right)}^{*}$ denotes the cost function which makes model learn features of the dataset, a quadratic cost function described as $J{\left(\theta ;X,y\right)}^{*}=\frac{1}{2B}\sum_{x}{∥y-\hat{y}\left(x\right)∥}^{2}$ is generally used, x∈X. $\hat{y}$ is the output of the network. *B* is the spectral number of the HSI dataset.

The DBN network is built as an auto-encoder which acts as an encoder at first and then as a decoder. Its output is expected to be the same as its input during the training. For this aim, the cost function is used to minimize the difference between inputs and the outputs. The pixels in *X* are not only fed to input layer as input samples but also used to replace the labels in the cost function for DBN network parameters updating. Because no extra labels are required, the training is in an unsupervised way.

The aim of training is to figure out the connection weight parameters *W* and the layer bias parameters *b* by the cost function of Equation (2). Gradient descent [38] training method is generally used for training. Every single pixel in the *X* is used as an independent sample to train the network one by one with all of the selected bands. For each sample, *W* and *b* are updated as Equation (3) and Equation (4) according to Reference [38].

(3) $$W^{\left(i\right)}=W^{\left(i\right)}-\beta \gamma^{\left(i\right)}h^{\left(i-1\right)}$$

(4) $$b^{\left(i\right)}=b^{\left(i\right)}-\beta \gamma^{\left(i\right)}$$

where $W^{\left(i\right)}$, $b^{\left(i\right)}$ and $h^{\left(i-1\right)}$ have the same meaning as in Equation (1). β is the learning rate of the network. Many methods [37,39,40] have been documented to determine β, and we set it to 0.3. γ denotes the residual of each layer, and it is described as $\gamma =\frac{\partial }{\partial z}J\left(\theta ;X,y\right)$, $z=W^{(i)T}h^{\left(i-1\right)}+b^{\left(i\right)}$.

After the training, the network can encode the input pixels as sparse code which contains the distribution feature of the input pixel and features of image dataset. The sparse code is gathered from the output of the middle layer [38] and regarded as Image Code (*C*). For each pixel, the Reconstruction Error is computed by Equation (5).

(5) $$r={\left(\sum_{i=1}^{B}{\left|x_{i}-h_{i}\right|}^{2}\right)}^{\frac{1}{2}}$$

where *r* denotes the reconstruction error of each pixel, r∈R. *B* denotes the total spectral bands number of the input dataset. *x* is the input pixel in *X* and *h* is the decoded output from the output layer. In this paper, *h* is equal to $\hat{y}$.

Due to low occurrence probability, anomalous pixels are far less than normal background pixels. Thus, anomalous pixels perform low contribution during training DBN model. Therefore, the model cannot learn the features of anomalous pixels well, and cannot describe anomalous pixels precisely. Thus during detection, the reconstruction errors of anomalous pixels are usually larger, which can be used for anomalousness detecting. However, if only the reconstruction errors are used to determine anomaly targets, due to some of the pixel information may be lost, it is hard to get high accuracy. So in the proposed method, the reconstruction information is used together with Image Code (*C*) to find anomaly targets.

### 3.2. The Framework of Proposed Method

**Figure 2:**
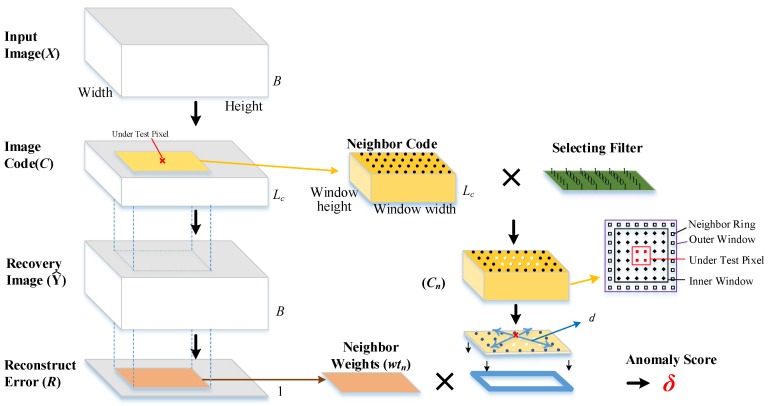
The data flow of the proposed algorithm, Windowheight and Windowwidth stand for the height and width of the local image window, respectively. *B* stands for spectral band number. $L_{c}$ stands for the length of the code. δ stands for the anomaly score.

Considering anomalies have lower occurrence probability than the background pixels, the reconstruction errors are directly used as anomaly score in DBN-AD [30]. To improve the detection accuracy, the local spatial information and the features in codes are engaged in the proposed method, the reconstruction errors are employed together with codes which are made up with $L_{c}$ outputs of $L_{c}$ neurons in middle layers of the network. Each code with $L_{c}$ independent values represent the features of each pixel of *X*. The distance between the code of under test pixels and its neighboring pixels is computed as anomaly score to discriminate anomalies. To reduce the adverse effect on distance computing from probable anomaly pixels, the reconstruction errors are organized as weights to adjust the importance of different pixels. The details of the proposed algorithm are stated in Algorithm 1, and the data flow of the proposed algorithm in detection stage is illustrated in Figure 2 with the following six steps:

Step1. Train the DBN model in an unsupervised way with Input Image (*X*) which is constructed with all the under test pixels.
Step2. Feed Input Image (*X*) to DBN model to generate the Image Code (*C* ) and Reconstruction Error (*R*). The *C* is generated from the output of the middle layer neurons. *R* is the differences between *X* and Recovery image ($\hat{Y}$) which is the decoded data array of *C* by DBN model.
Step3. Select neighboring pixels from the surrounding of under test pixel in *C*.
Step4. Calculate the distances between neighboring pixels code $c_{n}$ and the under test pixel code $c_{t}$ in *C*.
Step5. Calculate the neighbor weights $wt_{n}$ by Reconstruction Error (*R*).
Step6. Calculate the anomaly score δ by the neighbor weights $wt_{n}$ and the distances.

**Algorithm 1** Adaptive Weight DBN Based HSI Anomaly Detection**Input:** *X* HSI image dataset$N_{X}$ the number of pixels in *X*$N_{n}$ the number of selected neighboring pixels**Output:** δ Detection results that anomaly score   1: **function**
Anomaly Detection(*X*)   2:  $M_{DBN}$ ← Training via gradient descent with *X*   3:  (*C*, *R*) ← EncodeDecode($M_{DBN},X$)   4:  **for** j = 1 to $N_{X}$
**do**   5:    $c_{n}$ ← from *C* following Section 3.3   6:    $r_{n}$ ← from *R* following Section 3.3   7:    **for**
i=1 to $N_{n}$
**do**   8:      $wt_{n}\left[i\right]$ ← Equation (9) and Equation (10) with $r_{n}$   9:      *i* ← i+1 10:    **end for** 11:    δ[j] ← Equation (7) by using $wt_{n}$ and $c_{n}$ 12:    *j* ←j+1 13:  **end for** 14:  **return**
δ 15: **end function** 16: 17: **function**
EncodeDecode($M_{DBN},X$) 18:  Initialize the auto_encode from $M_{DBN}$ 19:  **for** j = 1 to $N_{X}$
**do** 20:    *x* ← one pixel from *X* 21:    Encode *x* with auto_encode 22:    C[j] ← output of middle layer of auto_encode 23:    $\hat{Y}\left[j\right]$ ← decode C[j] with auto_encode 24:    R[j] ← Equation (5) with h[j] and *x* 25:    *j* ← j+1 26:  **end for** 27:  **return**
*R* and *C* 28: **end function**

The DBN auto-encoder is trained before detection with HSI dataset *X*. Through this encoder, a code with lower dimension than spectral band number is generated for each pixel to form the *C*. During the detection, the pixels of *X* are fed to $M_{DBN}$ one by one. Only one pixel is encoded and then decoded by the $M_{DBN}$ at a time. During encoding, only one code is contained in the Code Layer of $M_{DBN}$. After the whole image input fed into the network, a code image can be got which is constructed by the codes. The code can be regarded as a feature transformation from original data space to the code space of the input pixel. Thus, the neighborhood of neighbor pixels in the original image is the same with the neighborhood of the codes which map to neighbor pixels in the Code image *C*. During the encoding, the decoding is run at the same time and generates a recovery image $\hat{Y}$ with the same band number as *X*. The difference between recovery image $\hat{Y}$ and input image *X* is used to produce the reconstruction error *R*. A pixel with a large *r* is more likely to be an anomaly pixel. In local based anomaly detection methods, if an anomaly pixel is contained in the selected neighboring pixels dataset, the detector may be contaminated. To weaken the impact of such anomalous pixels, adaptive weights wt computed by the reciprocal of reconstruction errors are used to modify neighbor distance δ.

### 3.3. Proposed Adaptive Weight-Based HSI Anomaly Detector

In deep auto-encode models for HSI feature learning, the reconstruction errors and code image can be generated. To well use the features extracted from the image by the DBN auto encoder in code layer, the Euclidean distance *d* between neighboring pixels code $c_{n}$ and the under test pixel code $c_{t}$ ($c_{n},c_{t}\in C$) is calculated. For *j*-th neighboring pixel, the distance is defined by Equation (6).

(6) $$d_{j}={\left(\sum_{i=1}^{L_{c}}{\left|c_{n}\left[j\right]\left[i\right]-c_{t}\left[i\right]\right|}^{2}\right)}^{1/2}$$

where $L_{c}$ is the dimension of pixel code, $c_{n}\left[j\right]\left[i\right]$ and $c_{t}\left[i\right]$ denote the code value of the *i*-th dimension in *j*-th local pixel and under test pixel, respectively.

To reduce the anomalous pixels contamination for a better detection, the contribution of anomalous pixels should be decreased. So we proposed a weighted distance in Equation (7) which can not only measure the similarity between under test pixel and its neighboring pixels but also modify the contribution of possible anomalous pixels.

(7) $$\delta =1/N_{n}\sum_{j=1}^{N_{n}}wt_{n}\left[j\right]d_{j}$$

where $N_{n}$ represents the number of neighboring pixels, $wt_{n}\left[j\right]$ represents the contribution of neighboring pixels.

**Figure 3:**
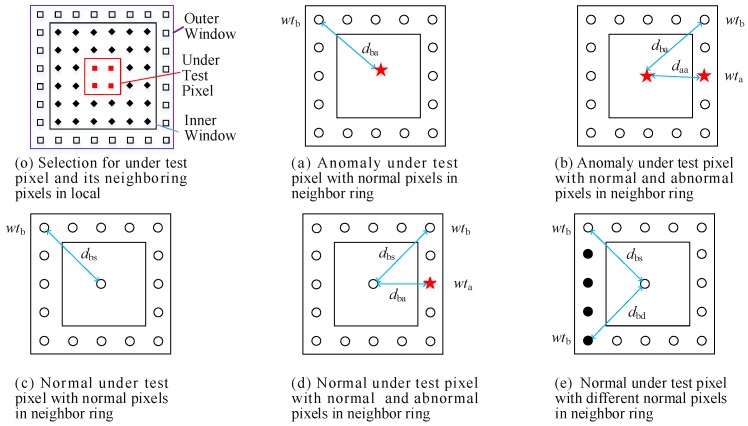
Different situations of under test pixels and the neighboring pixels

To select local pixels from the surrounding of under test pixel, a dual window is built up as shown in Figure 3o. The dual window covers the under test pixels, marked as “▉” and its surrounding pixels (which include some central pixels marked as “◆” and the neighboring pixels marked as “☐” between the outer window and inner window). In some situations, anomaly objects may occupy several pixels. To minimize false detection risk, central pixels “◆” are excluded, and only the neighboring pixels “☐” (named neighbor ring in Figure 3) are employed for Equation (7). In general, the window size should be larger than the expected anomalies. Before the detection, a window size needs to be determined depending on the size of expected anomaly targets. A bigger window size leads to more neighboring pixels being involved in distance computation (Equation (7)), thus more time is required for detection.

After neighboring pixels selection by the window, the codes selection is done as well. Because the locations of the neighboring pixels in *X* are the same with the locations of its neighboring pixel code in *C*. Because the reconstruction error of anomalous pixel is big, the weights of local neighboring pixels should have an opposite trend to its reconstruction errors. For example, a pixel whose reconstruction errors is large should be allocated a small weight. However, to get a higher precision, the weight of potential anomaly pixels and the distance between probable same background pixels should be controlled more finely. The following five distribution conditions in Figure 3a–e are more common in real HSI.

In Figure 3 , “○” denotes background class A, “•” denotes background class B, and “★” denotes anomalous pixels. The distance for the situations presented in Figure 3a–e can be, respectively, defined using Equation (8a–e).

(8a) $$\delta_{a}=\sum_{i=1}^{N_{n}}\left(d_{ba_{i}}\cdot wt_{b_{i}}\right)$$

(8b) $$\delta_{a}=\sum_{i=1}^{M}\left(d_{ba_{i}}\cdot wt_{b_{i}}\right)+\sum_{j=1}^{K}\left(d_{as_{j}}\cdot wt_{a_{j}}\right)$$

(8c) $$\delta_{b}=\sum_{i=1}^{N_{n}}\left(d_{bs_{i}}\cdot wt_{b_{i}}\right)$$

(8d) $$\delta_{b}=\sum_{i=1}^{P}\left(d_{ba_{i}}\cdot wt_{a_{i}}\right)+\sum_{j=1}^{Q}\left(d_{bs_{j}}\cdot wt_{b_{j}}\right)$$

(8e) $$\delta_{b}=\sum_{i=1}^{O}\left(d_{bs_{i}}\cdot wt_{b1_{i}}\right)+\sum_{j=1}^{R}\left(d_{bd_{j}}\cdot wt_{b2_{j}}\right)$$

where $\delta_{a}$ is the distance of an anomaly pixel and its neighboring pixels. $\delta_{b}$ is the distance of a normal pixel and its neighboring pixels. $\delta_{a}$ and $\delta_{b}$ are the anomaly score. $N_{n}$ is the total number of neighboring pixels. *P* and *K* are the numbers of anomalous pixels in neighbor ring in Figure 3b,d, respectively. *M* and *Q* are the normal (background) pixel numbers of the neighbor ring in Figure 3b,d, respectively. *O* and *R* are the numbers for background class A pixels and background class B pixels of the neighbor ring in Figure 3e, respectively. $wt_{b}$, $wt_{b1}$ and $wt_{b2}$, which are the weights of normal neighbor pixels, are assumed to be large. $wt_{a}$ which is the abnormal neighboring pixel is assumed to be small. $d_{bs}$ and $d_{as}$, respectively, denote the distance between the same background pixels and the distance between anomaly pixels. They are supposed to be small values. $d_{ba}$ and $d_{bd}$, which, respectively, denote the distance between background pixels and anomalous under test pixels and the distance between different background pixels, are supposed to be big values.

To further improve detection accuracy, we propose a adaptive weight modification technique. Under the conditions shown in Figure 3a,b, the under test pixels are anomaly pixels. Thus, a large value of $\delta_{a}$ is expected for a better detection accuracy, while a small value of $\delta_{b}$ is expected in Figure 3c–e. By definition, anomalous pixels occur with low probability. Thus, in most real situations, *M* is much larger than *K*, and *Q* is much larger than *P*. The expected and the most likely occurrences are shown in Table 1.

In Table 1, “△” means that a big distance value is expected for better detection accuracy. “▽” means that a small distance value is expected for better detection accuracy. “▴” means that it is a big value for most of the situations. “▾” means that it is a small value for most of the situations.

In general, if $wt_{a}$ can be reduced, the value of $\delta_{b}$ can be further reduced to improve the detection accuracy. However, considering the condition in Figure 3b, the decrease of $wt_{a}$ may cause $\delta_{b}$ to decline which is not expected for better detection. However, in general, M>K, thus the impact on $\delta_{b}$ by the modification of $wt_{a}$ can be ignored. Thus, the key problem is how to identify $wt_{a}$ during detection. Considering the situation shown in Figure 3d, $wt_{a}$ is smaller and rarer than others neighboring pixels during detection. However, in traditional detectors, it is difficult to find the anomalous pixels and their corresponding $wt_{a}$ before detecting. In this paper, a statistical method is used to identify $wt_{a}$ by confidence level checking. Therefore, most of $wt_{a}$ can be further reduced by decreasing the neighboring pixels’ weights which fail to pass the check.

Then neighboring weight $wt_{n}$ is calculated by the reconstruction error $r_{n}$, $r_{n}\in R$. The difference between $r_{n}$ and its mean is compared with its standard deviation. If the difference is larger than the standard deviation, the weight of this neighboring pixel should be reduced by a penalty factor according to Equation (10). For other weights, they are computed by Equation (9).

(9) $$wt_{j}=\frac{1}{r_{j}}$$

(10) $$wt_{j}=Pf\cdot \frac{1}{r_{j}}$$

where $Pf\in \left(0⩽Pf⩽1\right)$ is the penalty factor.

Through Equations (7), (5), (9) and (10), the anomalies can be determined by the score of δ in Equation (7).

## 4. Experiments

### 4.1. Dataset

Two synthetic and a real HSI dataset are used to verify the proposed method.

**Figure 4:**
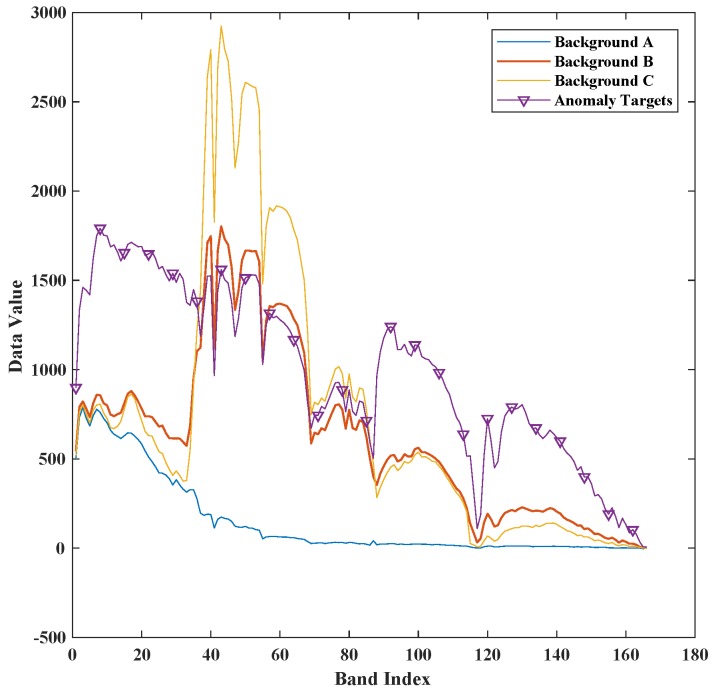
The spectral signature of anomaly targets and different background pixels in Lake Salvador dataset.

**Figure 5:**
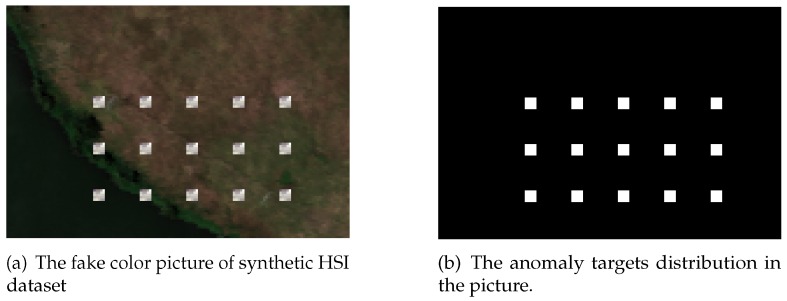
The HSI image of Lake Salvador dataset.

The first synthetic dataset is made of a real HSI dataset which was acquired on Lake Salvador using Airborne Visible Infrared Imaging Spectrometer (AVIRIS) [41] in September 2010. This real dataset was downloaded from NASA (http://aviris.jpl.nasa.gov/),file f100930t01p00r13. The bands with central frequency of 0.37 μm–0.38 μm, 0.90 μm –0.97 μm, 1.11 μm–1.16 μm, 1.33 μm–1.50 μm and 1.78 μm–1.98 μm [42,43,44], are water absorption and low signal-to-noise ratio bands. These bands are removed for better detection accuracy [45,46,47]. After removing the water vapor absorption spectral bands, 166 spectral bands are selected from 224. The anomaly targets are generated by embedding 15 building image blocks which are selected from city landscape in the same HSI image. The size of the image block is 5×5. Its spectral signature is shown in Figure 4. The ground truth file is recorded when embedding the anomaly blocks. The entire synthetic image and ground truth image is shown in Figure 5.

**Figure 6:**
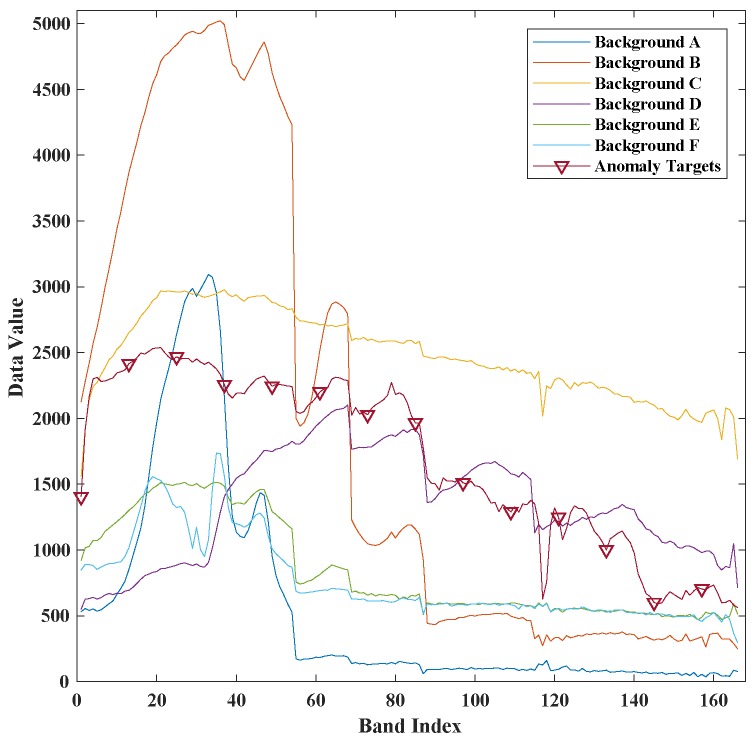
The spectral signature of anomaly targets and background pixels of San Jose.

**Figure 7:**
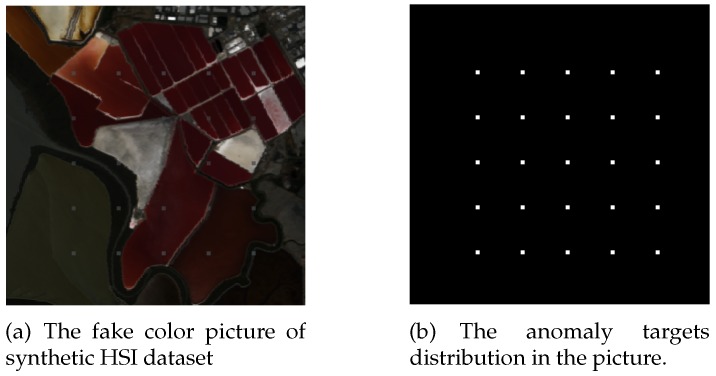
The HSI image of San Jose dataset.

The second synthetic dataset is recorded over San Jose, United States of America, in May 2014 by AVIRIS. An image with a size of 300×300 and 16.4 m spatial resolution is used for experiments. The dataset was downloaded from NASA, file f140528t01p00r10. After water absorption and low signal-to-noise ratio bands remove, 166 spectral bands from the wavelength of 0.4 μm–2.5 μm are taken for the detection. More kinds of backgrounds are used than first synthetic dataset, and the anomaly targets are embedded in a more complex way. A series blocks image with the size of 4×4 are generated by mixing 70% of a ship pixel and 30% of the background pixels which will be replaced by anomaly targets. Its spectral signature is shown in Figure 6. The second synthetic and the anomaly targets position are shown in Figure 7.

**Figure 8:**
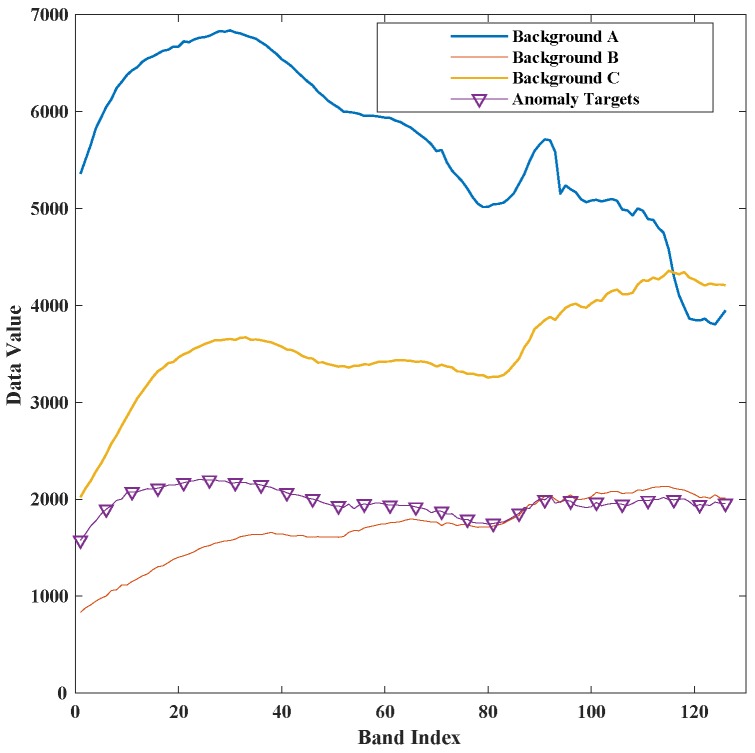
The spectral signature of anomaly targets and average background pixel of San Diego airport.

**Figure 9:**
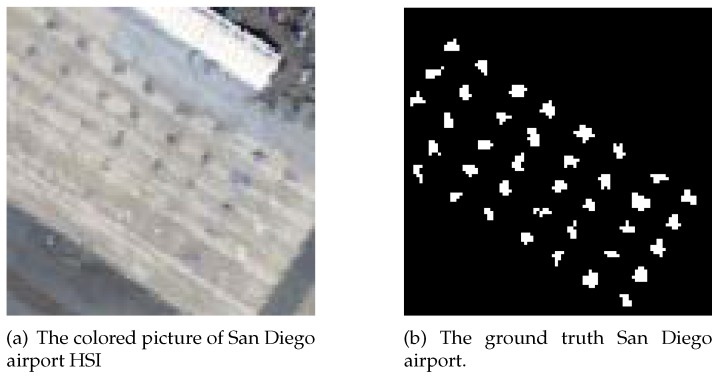
The real HSI image.

A real HSI dataset which contains ground truth labels of anomaly targets is also used to validate the proposed method. This dataset is collected by AVIRIS over the San Diego airport [26]. After removing the water vapor absorption and interference bands from the original 224 spectral bands (between 0.4 μm and 2.5 μm), 126 spectral bands are used for anomaly detection, with 38 planes as anomaly targets. The different background and planes spectral signature in San Diego airport dataset are shown in Figure 8. The portion selected for experiments is displayed in Figure 9a, while Figure 9b is the ground-truth label which contains the spatial location of the planes.

### 4.2. Experiment Environment and Evaluation Criteria

The experiments are run on an AMAX PSC-HB1X workstation which has two Intel Xeon Haswell E5-2640v4 processors using Matlab 2016b. To evaluate the proposed methods, the RXD and LRXD algorithms are used as baseline methods, and the state-of-the-art CRD method is also used for comparison. For estimating the contribution of proposed adaptive weight method, the DBN-AD [30] is run on the same dataset and same parameters with the proposed method. DBN-AD directly employs the reconstruction errors of every pixel as its anomaly score. To analyze the contribution of the proposed weight method, DBN local reconstruction errors based anomaly detector (DBN-LAD) is built up only from reconstruction errors. The anomaly score of DBN-LAD is the distance between the neighboring pixels code and the under test pixels code without the proposed adaptive weight. By comparing DBN-LAD with DBN-AD, the contribution of the code can be analyzed, and by comparing Proposed method to DBN-LAD, the contribution of adaptive weights can be analyzed. As some anomaly scores may be far higher than the others, a grayscale image that is gray-level transformed directly from anomaly score image may not be clear enough for displaying the results. To give the detection results, fixed false positive rate for each dataset is set. A pixel with distance score larger than the threshold should be considered as an anomalous pixel and shown as a white point in the target detection result image. Normal pixels are set to black.

Two criteria are used to evaluate the proposed methods and the other algorithms: (1) receiver operating characteristic (ROC) curve; and (2) the area under the ROC curve (AUC) [48]. To display the performance of different detectors at low False Positive Rate in ROC, the ROC figure is shown on a log scale.

### 4.3. Results and Discussion

In local anomaly detector, the local window size affects the detection accuracy. Generally, the local window is set larger than the expected anomaly objects [49]. The bigger the window size is, the longer detection executing time is required. Thus, a detector that can perform with a smaller size of the local window is more promising. In this study, the optimum local windows size are different for different detectors. Thus, to make a fair comparison, a window size searching is executed ranging from 6×6 (a little bigger than expected targets) to 40×40. For RXD, to avoid generating singular matrix during detection, the number of surrounding pixels should be larger than the number of spectral bands [50]. During the searching, a suitable window size which can make a detector produce a bigger AUC value is selected for the detector. After optimization on two synthetic datasets and the real HSI dataset, the window size of the proposed detector, DBN-LAD, LRXD and CRD are set as shown in Table 2. To evaluate the contribution of the proposed adaptive weight methods, the DBN-LAD is set in the same window size with proposed detector.

**Figure 10:**
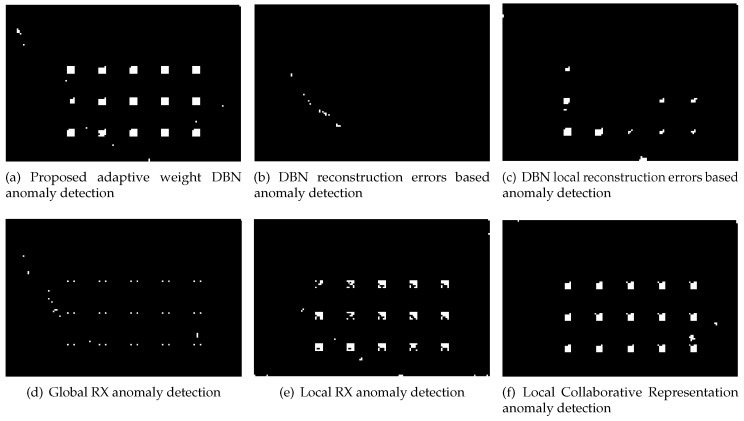
The targets detection result image of Lake Salvador HSI image. Fixed false positive rate for all images in Figure 10 is 0.001.

**Figure 11:**
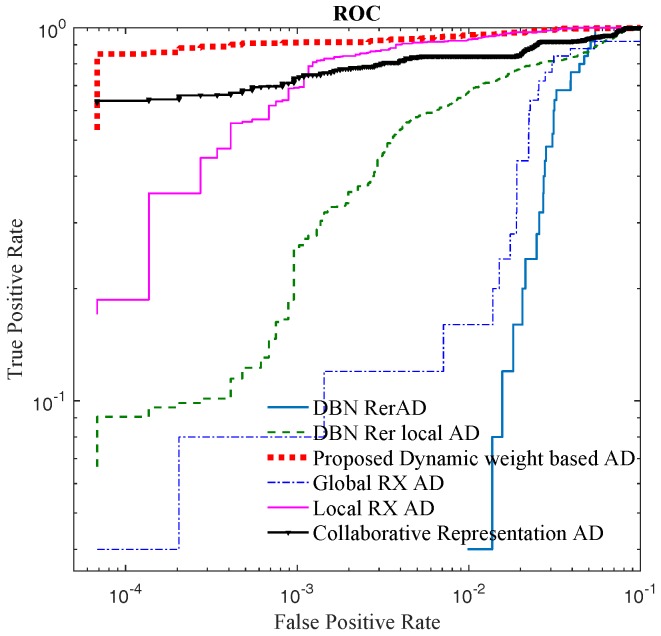
The ROC of Lake Salvador HSI image.

The synthetic image is firstly used to verify the proposed method. The results by the proposed method, DBN-AD, DBN-LAD, RXD, LRXD and CRD are shown in Figure 10. The ROC results are shown in Figure 11.

According to the ROC of Lake Salvador HSI image in Figure 11, the ROC curve of the proposed method is higher than the other detectors, and at low false positive rate, the proposed method performs well which is indicated by Figure 11 and Figure 10. In Figure 10, the proposed method and CRD can clearly indicate the location and the shape of embedded anomalous blocks at fixed false positive rate of 0.001. The AUC values, detection time and the training time of all the detectors are presented in Table 3. In Table 3, the AUC of the proposed algorithm, LRXD and CRD are in the same level, while the proposed detector is executed with less time consumption. In local based detectors, as the local pixels are processed independently when detecting different under test pixels, the computational burdens of local based detectors are heavier than the global based methods. Thus, the proposed detector has longer detection time than other global based detectors (RXD and DBN-AD). However, compared to Local RXD and CRD, the proposed detector is more efficient. Because DBN-AD just needs forward inference without distance computation, so DBN-AD has less detection time than proposed method and DBN-LAD. In training stage, the proposed method, DBN-LAD, DBN-AD, and DBN model and their parameters are the same. The difference is just in the computation of anomaly score. Thus, for the same dataset, the proposed method, DBN-LAD and DBN-AD have the same training time.

**Figure 12:**
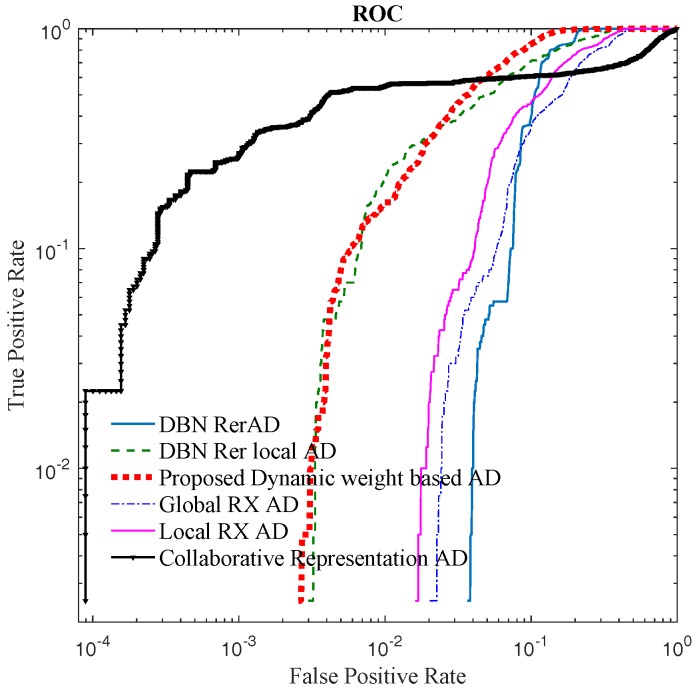
The ROC of San Jose HSI image.

**Figure 13:**
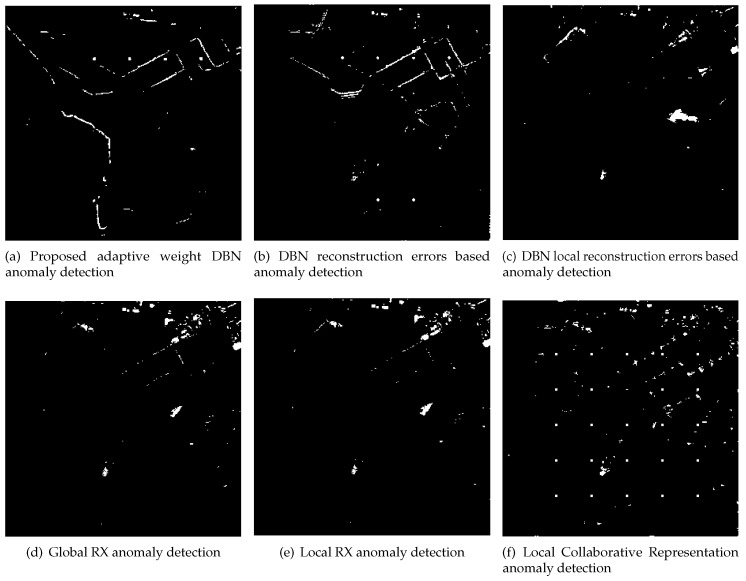
The targets detection result image of San Jose HSI image. Fixed positive alarm rate for all images in Figure 13 is 0.01.

For the synthetic HSI image of San Jose, according to the ROCs in Figure 12, when the false positive rate value is smaller than 0.03, the CRD can outperform other detectors which is indicated by Figure 13 as well (where the false positive rate is fixed to 0.01). When the false positive rate value is bigger than 0.03, the proposed method outperforms other detectors (Figure 12) in terms of ROC. The AUC results and detecting time are shown in Table 4 for different detectors on San Jose dataset. In Table 4, the proposed method outperforms other algorithms in term of AUC value. From the table, the proposed adaptive DBN detector outperforms DBN-LAD and DBN-AD in term of AUC value. It mainly benefits from the contribution of adaptive weights which improve the AUC from 0.915 (DBN-LAD) to 0.949 (proposed method), while about 18 more seconds are consumed. Considering on detecting time, the global based detectors run faster than local based methods. As local based detector, the DBN based methods need about 13.278 s for model training in the San Jose dataset, and the proposed adaptive DBN detector is over ten times more efficient than LRXD and CRD.

**Figure 14:**
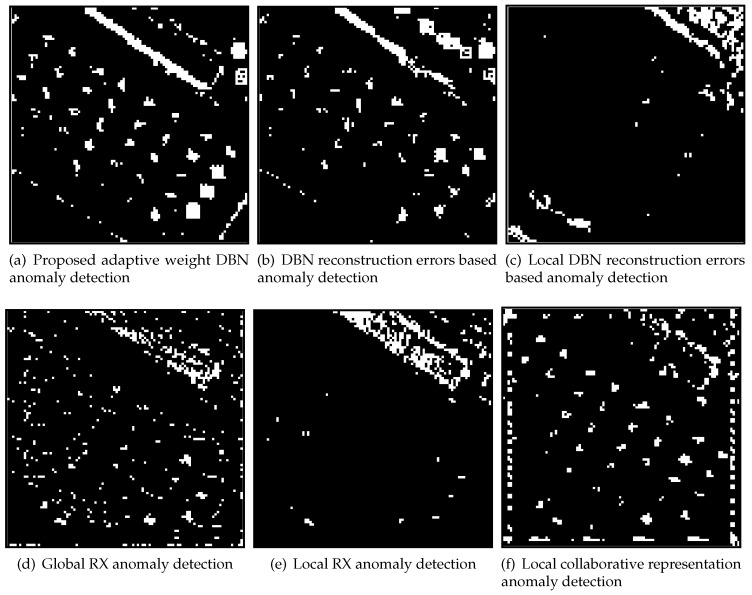
The targets detection results image of San Diego airport HSI image. Fixed false positive rate for all images in Figure 14 is 0.05.

**Figure 15:**
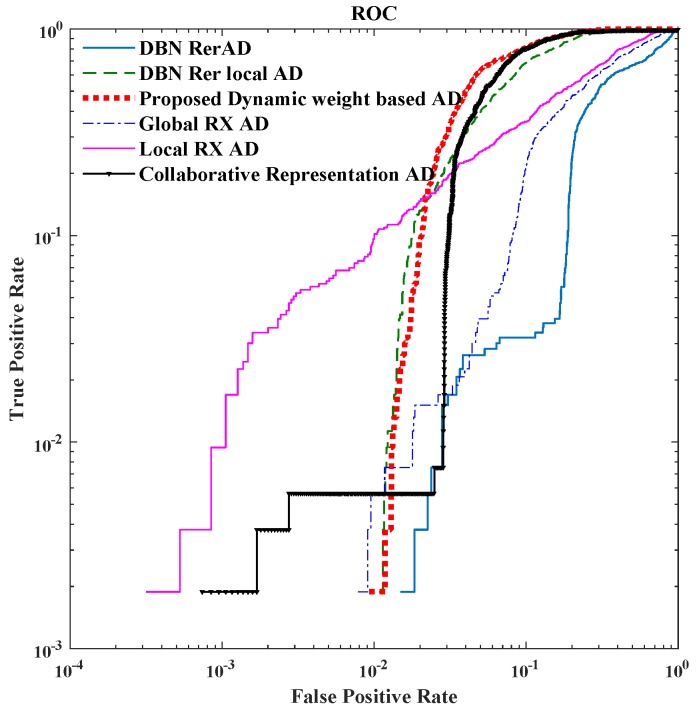
The ROC of San Diego airport HSI image.

For the real HSI dataset, the detection result images and the ROC are shown in Figure 14 and Figure 15, respectively.

**Figure 16:**
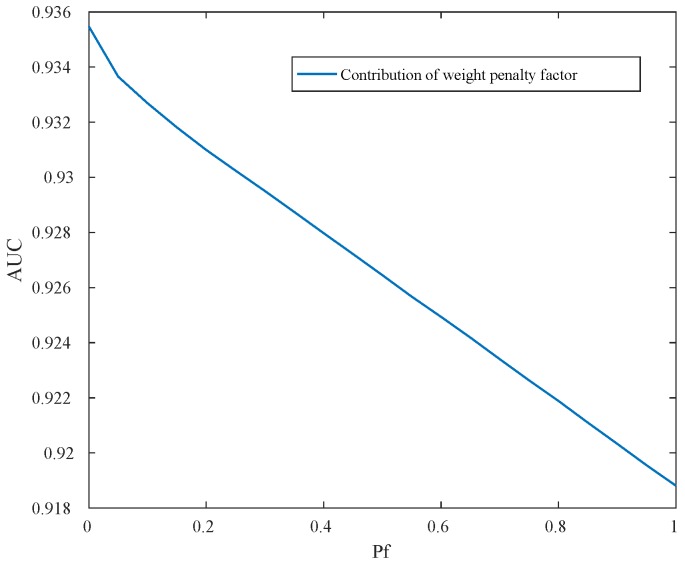
The effect of the weight penalty factor, Pf, in anomaly detection for San Diego airport HSI image.

For the real image dataset, in Figure 15 and Table 5, the proposed detector has greater AUC value and is almost 14× faster than CRD. When FPR is lower than 0.01, the local RXD performs well, and the proposed method is not superior to others. When FPR is over 0.01, the proposed detector performs well which is indicated in Figure 14 as well. In Figure 14, when FPR is fixed to 0.05, the proposed detector shows the shape and position of anomaly targets clearly. The maximum AUC for the above image is 0.87 when only the reconstruction errors (in DBN-AD) are used from a dedicated DBN network. In DBN-LAD, a higher AUC is obtained by adding a local detection strategy and using the code for the distance calculating, increasing the AUC to 0.907. With the help of adaptive weights, the AUC can reach 0.935, which is a dramatic improvement. Comparing to DBN-LAD, the adaptive weights strategy contribute to the detection accuracy improving. From the proposed Equation (10), a small value of weight penalty factor is expected when the weights correspond to the anomaly pixels. The relationship between AUC value and the weight penalty factors are shown in Figure 16. As the penalty factor is increased from 0 to 1, the AUC value decreases from 0.935 to 0.919 in an almost linear fashion. Thus, the penalty factor is recommended to set to 0.

## 5. Conclusions

In this paper, an adaptive weight DBN based anomaly detection method was proposed for hyperspectral imagery. It used an unsupervised auto-encoder to learn the high-level features of the HSI dataset. To overcome contamination by abnormal pixels, a statistic method was proposed to identify the potential local anomaly pixels and reduce their impact via penalty factors. By combining the reciprocal of the reconstruction errors and the penalty factors, adaptive weights were used to measure the contribution of the neighboring pixels. The effects of penalty factors and proposed adaptive weight DBN method on the performance of DBN reconstruction errors were also analyzed. Experimental results demonstrate that, after local window size optimization, the proposed method outperforms the existing RXD, LRXD and state-of-the-art CRD in term of AUC.

Our technique could be further improved in terms of computational efficiency and the detection performance at low false positive rate.

## Figures and Tables

**Table 1:** The expected value and the relationship of distance, neighboring pixels and weights.

Distance Name	Expected Trend	Distance Type	Weight	Distance Type	Weight
$\delta_{a}$ in Equation (8a)	△	$d_{ba}▴$	$wt_{b}▴$	-	-
$\delta_{a}$ in Equation (8b)	△	$d_{ba}▴$	$wt_{b}▴$	$d_{as}▾$	$wt_{a}▾$
$\delta_{b}$ in Equation (8c)	▽	$d_{bs}▾$	$wt_{b}▴$	-	-
$\delta_{b}$ in Equation (8d)	▽	$d_{bs}▾$	$wt_{b}▴$	$d_{ba}▴$	$wt_{a}▾$
$\delta_{b}$ in Equation (8e)	▽	$d_{bs}▾$	$wt_{b1}▴$	$d_{bd}▴$	$wt_{b2}▴$

**Table 2:** Window size for different detectors in different dataset after optimization.

Dataset Name	Window Size of Proposed Detector & DBA-LAD	Window Size of LRXD	Window Size of CRD
San Diego airport	8×8	28×28	outer window: 14×14, inner window: 8×8
Lake Salvador	8×8	30×30	outer window: 16×16, inner window: 10×10
San Jose	6×6	22×22	outer window: 18×18, inner window: 8×8

**Table 3:** AUC values and processing time of different methods for the Lake Salvador HSI image.

Method	AUC Value	Detection Time (s)	Training Times (s)
Proposed adaptive weight DBN Detector	0.998	19.510	3.812
Local Reed-Xiaoli Detector [7]	0.998	66.455	-
Collaborative Representation Detector [16]	0.993	41.174	-
DBN local reconstruction errors Detector	0.985	3.361	3.812
Global Reed-Xiaoli Detector [6]	0.972	0.306	-
DBN-AD [30]	0.968	0.435	3.812

**Table 4:** AUC values and processing time of different methods for the synthetic HSI image of San Jose.

Method	AUC Value	Detection Time (s)	Training Times (s)
Proposed adaptive weight DBN Detector	0.949	26.424	13.278
DBN local reconstruction errors Detector	0.915	8.722	13.278
DBN-AD [30]	0.885	0.143	13.278
Local Reed-Xiaoli Detector [7]	0.858	284.978	-
Global Reed-Xiaoli Detector [6]	0.820	1.423	-
Collaborative Representation Detector [16]	0.762	558.823	-

**Table 5:** AUC values and processing time of different methods for the San Diego airport HSI image dataset.

Method	AUC Value	Detection Time (s)	Training Times (s)
Proposed adaptive weight DBN Detector	0.935	2.483	3.58
Collaborative Representation Detector [16]	0.917	34.380	-
DBN local reconstruction errors Detector	0.907	1.511	3.58
DBN-AD [30]	0.870	0.985	3.58
Local Reed-Xiaoli Detector [7]	0.776	26.682	-
Global Reed-Xiaoli Detector [6]	0.698	0.150	-

## Abbreviations

The following abbreviations are used in this manuscript:

AD	Anomaly Detector
AUC	Area Under the ROC Curves
AVIRIS	Airborne Visible Infrared Imaging Spectrometer
CRD	Collaborative Representation Detector
DBN	Deep Belief Network
FPR	False Positive Rate
HSI	Hyperspectral Image
LRXD	Local RX Detector
ROC	Receiver Operating Characteristic Curve
RXD	Reed-Xiaoli Detector
TPR	True Positive Rate
